# Cyto-genotoxicity and immunomodulation in fibroblasts exposed to calcium-aluminate cement with different radiopacifiers

**DOI:** 10.1590/0103-644020256511

**Published:** 2025-12-01

**Authors:** Antônio Secco Martorano, Nadyne Saab Messias, Rayana Longo Bighetti-Trevisan, Lucas Novaes Teixeira, Ivone Regina de Oliveira, Walter Raucci-Neto, Paulo Tambasco de Oliveira, Larissa Moreira Spinola de Castro-Raucci

**Affiliations:** 1 School of Dentistry of Ribeirão Preto, University of São Paulo, Ribeirão Preto, SP, Brazil.; 2 School of Dentistry, University of Ribeirão Preto, Ribeirão Preto, SP, Brazil; 3 Cell Biology and Oral Pathology Division, Faculdade São Leopoldo Mandic, Campinas, SP, Brazil; 4 Institute for Research and Development, University of Vale do Paraíba, São José dos Campos,SP, Brazil

**Keywords:** DNA Damage, Endodontics, Fibroblast, Gene Expression, Inflammation

## Abstract

Cimento de aluminato de cálcio (CAC) tem sido proposto como alternativa ao agregado de trióxido mineral (MTA), porém seu comportamento biológico permanece pouco explorado. Este estudo avaliou a citotoxicidade, a genotoxicidade e a expressão de genes pró-inflamatórios em fibroblastos expostos a formulações de CAC contendo óxido de zinco ou óxido de bismuto, com diferentes proporções de cloreto de cálcio, em comparação ao MTA. Os fibroblastos foram cultivados com CACz (25% ZnO + 2,8% CaCl₂), CACb (25% Bi₂O₃ + 2,8% CaCl₂), CACb+ (25% Bi₂O₃ + 10% CaCl₂) ou MTA; células não expostas serviram como controles. A citotoxicidade foi avaliada por morfologia em fluorescência e ensaio de viabilidade MTT em 24 h e 72 h; a genotoxicidade, pelo ensaio do cometa em 24 h; e a expressão gênica de COL-1, TNF-α, IL-1β, IL-6 e MMP-9 por RT-qPCR em 72 h. Os dados foram analisados pelo teste de Kruskal-Wallis, seguido do pós-teste de Student-Newman-Keuls (α = 0,05). A viabilidade celular não diferiu entre os grupos em 24 h, mas foi significativamente maior para o CACb+ em 72 h (p < 0,05). Os níveis de dano ao DNA foram semelhantes entre os grupos (p > 0,05). Quanto à expressão gênica, não foram observadas diferenças significativas para COL-1, IL-1β ou TNF-α. Todos os cimentos aumentaram significativamente a expressão de IL-6 em comparação ao controle (p < 0,05), com os maiores níveis no MTA e os menores no CACb+. O CACb+ aumentou significativamente a expressão de MMP-9 (p < 0,05), enquanto o CACz apresentou os menores níveis. Dentro das limitações deste estudo, as formulações de CAC com aditivos não foram genotóxicas nem citotóxicas, e seu comportamento biológico foi dependente da composição, sendo que o CACb+ demonstrou o perfil mais favorável ao potencializar a viabilidade de fibroblastos e o remodelamento da matriz extracelular.

## Abstract (en)

Calcium aluminate cement (CAC) has been proposed as an alternative to mineral trioxide aggregate (MTA), but its biological behavior remains underexplored. This study evaluated the cytotoxicity, genotoxicity, and proinflammatory gene expression of fibroblasts exposed to CAC formulations containing zinc oxide or bismuth oxide with different proportions of calcium chloride, in comparison with MTA. Fibroblasts were cultured with CACz (25% ZnO + 2.8% CaCl₂), CACb (25% Bi₂O₃ + 2.8% CaCl₂), CACb+ (25% Bi₂O₃ + 10% CaCl₂), or MTA; non-exposed cells served as controls. Cytotoxicity was assessed by fluorescence microscopy and MTT viability assays at 24 h and 72 h, while genotoxicity was evaluated by the comet assay at 24 h. Gene expression of COL-1, TNF-α, IL-1β, IL-6, and MMP-9 was examined by RT-qPCR at 72 h. Data were analyzed using the Kruskal-Wallis test and the Student-Newman-Keuls post hoc test (α = 0.05). Cell viability did not differ among groups at 24 h, but was higher for CACb+ at 72 h (p < 0.05). DNA damage levels were similar across groups (p > 0.05). For gene expression, no significant differences were observed for COL-1, IL-1β, or TNF-α. All cements upregulated IL-6 compared with control (p < 0.05), with the highest levels in MTA and the lowest in CACb+. CACb+ increased MMP-9 expression (p < 0.05), while CACz showed the lowest levels. Within the limits of this study, CAC formulations with additives were not genotoxic or cytotoxic, and their biological behavior was composition-dependent, with CACb+ demonstrating the most favorable profile by enhancing fibroblast viability and extracellular matrix remodeling potential.

**Figure:**
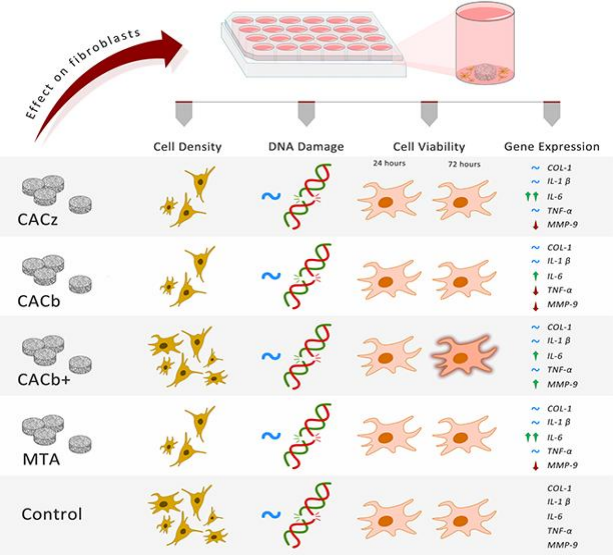

## Introduction

Calcium aluminate cement (CAC) is a hydraulic cement developed for endodontic purposes ^1^. Previous studies have demonstrated that this cement possesses satisfactory physicochemical properties, antimicrobial activity, and a low production cost, positioning it as a potential alternative to mineral trioxide aggregate (MTA), which is currently regarded as the gold standard in endodontics ^2^.

Mineral trioxide aggregate (MTA) and other calcium silicate-based cements (CSC) are widely used in endodontics due to their excellent sealing ability, biocompatibility, and bioactivity ^2^. However, these materials present inherent limitations, such as extended setting time, handling difficulties, high cost, and susceptibility to discoloration, particularly in formulations containing bismuth oxide ^3^. In this context, calcium aluminate cement (CAC) emerges as a promising alternative. Compared to CSCs, CAC exhibits superior mechanical strength, reduced solubility, and enhanced chemical stability ^4^. Additionally, CAC demonstrates a faster setting when modified with accelerators, such as calcium chloride, and has shown comparable or even superior biological properties in terms of biocompatibility, antimicrobial activity, and the promotion of mineralized tissue repair ^5^ ^,^ ^6^. Therefore, developing and validating CAC-based formulations aims not only to overcome the drawbacks of CSCs but also to provide cost-effective and biologically favorable alternatives for endodontic applications.

To improve CAC’s clinical performance, additives such as bismuth oxide and calcium chloride have been incorporated. Bismuth oxide is commonly used to confer adequate radiopacity, meeting international standards ^3^; however, some studies have raised concerns about its potential to interfere with biological responses, depending on concentration and interaction with the cement matrix ^6^. Calcium chloride, widely employed as a setting accelerator, has been shown to improve handling, reduce setting time, and modulate biological behavior by influencing ion release and alkalinity ^5^. Despite the known impact of these additives on physical and chemical properties, their biological effects, particularly with respect to genotoxicity and inflammatory modulation, remain largely unelucidated.

Several studies have investigated the bioactivity of CAC, demonstrating its capacity to stimulate the expression of genes associated with osteogenic differentiation (Runx2, Osterix, alkaline phosphatase, bone sialoprotein, and osteocalcin) and odontogenic differentiation (dentin matrix protein 1) ^5^ ^,^ ^6^, as well as to promote mineralized tissue formation ^1^. However, most of these investigations have focused on cell types with osteoblastic or odontoblastic potential. The biological response of fibroblasts, which are essential for maintaining connective tissue integrity and play a role in modulating inflammatory and healing processes in periapical tissues, remains poorly understood, particularly in response to CAC formulations containing bismuth oxide and varying concentrations of calcium chloride.

Furthermore, although cytocompatibility assessments of CAC are relatively well documented ^4^ ^,^ ^6^, there is a critical gap concerning its genotoxic potential. Genotoxicity assessment is crucial for materials intended for direct contact with periapical tissues, since DNA damage may impair tissue healing through mechanisms such as oxidative stress, apoptosis, or chronic inflammation ^7^. In fact, various dental materials have been associated with oxidative stress and the release of proinflammatory cytokines, such as interleukin-1β (IL-1β) and tumor necrosis factor-alpha (TNF-α), which are known contributors to the persistence and progression of periapical lesions ^8^.

Despite the promising physicochemical and biological properties reported for CAC, the scientific literature lacks comprehensive data regarding the biological safety of CAC formulations modified with bismuth oxide and calcium chloride. While previous studies have primarily focused on osteogenic or odontogenic cells, there is a critical gap in understanding how these modified cements interact with fibroblasts, which play an essential role in soft tissue healing and inflammatory modulation in the periapical environment. Although no genotoxicity or cytotoxicity was observed in murine fibroblasts exposed to Portland cement containing 15 % bismuth oxide ^9^, other investigations demonstrated that bismuth released from ProRoot MTA over 180 days reduced fibroblast viability and altered gene expression, including the upregulation of metallothioneins and downregulation of Col-1a and BSP ^10^. Additional findings suggest that incorporating bismuth oxide may increase cytotoxicity compared to unmodified cement ^11^ and that Bi₂O₃ nanoparticles can induce oxidative stress-mediated toxicity in endothelial cells at low concentrations ^12^. Moreover, the potential genotoxic effects of CAC formulations incorporating these additives have not been adequately investigated. Given that bismuth compounds have been associated with variable cytocompatibility outcomes and that calcium chloride significantly alters the material’s ionic release and setting behavior, it is crucial to determine whether these modifications influence DNA integrity and the expression of inflammatory mediators.

In this context, we hypothesized that incorporating different additives into CAC could modulate cellular responses and inflammatory activity, thereby contributing to improved tissue repair. The null hypothesis was that different calcium aluminate cement formulations would not affect fibroblast viability, genotoxicity, or the expression of proinflammatory genes. This hypothesis was tested by evaluating the cytotoxic and genotoxic effects of these cements, as well as their influence on gene expression related to extracellular matrix production and inflammation.

## Materials and methods

### Cell culture

**Figure 1:**
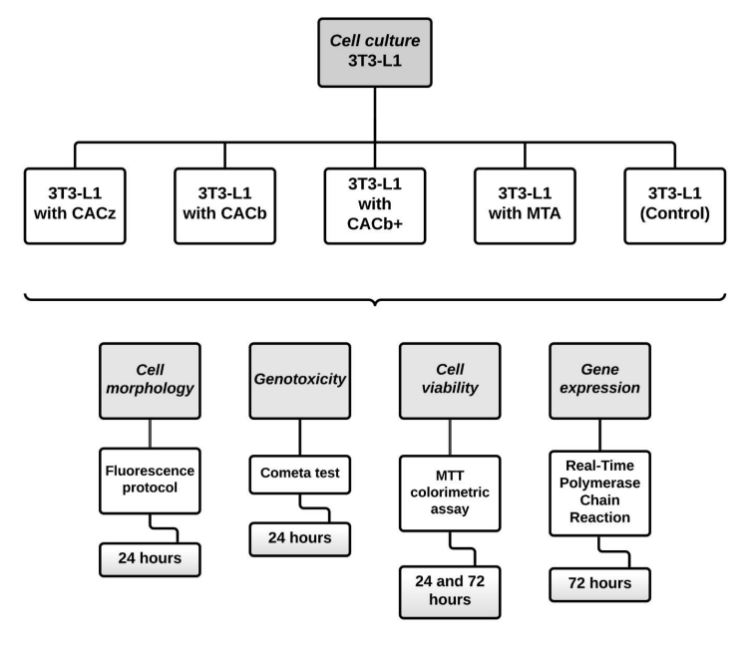
Flowchart representing the experiments performed with fibroblastic lineage 3T3-L1 cultured with different cement preparations.

All of the experiments described in the Materials and Methods section are illustrated in Figure 1. Cells from mouse fibroblastic lineage 3T3-L1 (American Type Culture Collection, Manassas, USA) were cultured in 75 cm^2^ culture flasks (Corning, New York, USA) with Dulbecco’s Modified Eagle Medium (DMEM) culture medium (Thermo Fisher Scientific, Waltham, USA), 10% fetal bovine serum (Thermo Fisher Scientific), 100 μg/ml streptomycin and 100 IU/ml penicillin (Thermo Fisher Scientific). Cells were maintained at 37°C in a humidified atmosphere containing 5% CO_2_ and 95% atmospheric air, and the medium was replenished every 2-3 days. After confluence, cells were plated in 24-well plates at 1 × 10^^4^ cells/well and left to adhere for 24 h prior to exposure to the cements. The cell morphology assay was performed by plating cells onto Thermanox^®^ coverslips (Nunc, Rochester, USA).

### Sample preparation

The following cements were used: ^1^ CACz, containing 25% w/w ZnO and 2.8% w/w CaCl₂; ^2^ CACb, containing 25% w/w Bi₂O₃ and 2.8% w/w CaCl₂; ^3^ CACb+, containing 25% w/w Bi₂O₃ and 10% w/w CaCl₂; and ^4^ MTA (Angelus, Londrina, Brazil). The chemical composition of each material is presented in Table 1. MTA was prepared according to the manufacturer’s instructions, while the CAC formulations were manipulated with deionized water (powder-to-liquid ratio 3:1 v/v) ^4^. Cylindrical specimens (4 mm in diameter and 3 mm in height) were prepared as described elsewhere ^5^ and allowed to set for four hours before being placed in contact with the cell culture. Considering that the initial setting time of these materials is approximately 20 minutes, a four-hour period was chosen to ensure complete hardening and to minimize the release of potentially cytotoxic substances present during the initial hydration reaction, thereby providing a more stable material surface for testing ^13^. The specimens were then placed in direct contact with adherent cells for 24 h, following ISO 10993-5 recommendations for direct contact assays.

**Table 1:** Chemical composition of the tested cements.

Cements	Composition (%)
CACz	Al_2_O_3_ (>68.5); CaO (<31); SiO_2_ (<0.8); Fe_2_O_3_ (<0.4); CaCl_2_ (2.8); ZnO (25)
CACb	Al_2_O_3_ (>68.5); CaO (<31); SiO_2_ (<0.8); Fe_2_O_3_ (<0.4); CaCl_2_ (2.8); Bi₂O₃ (25)
CACb+	Al_2_O_3_ (>68.5); CaO (<31); SiO_2_ (<0.8); Fe_2_O_3_ (<0.4); CaCl_2_ (10); Bi₂O₃ (25)
MTA	SiO_2_ (18.58); CaO (49.20); Al_2_O_3_ (4.48); MgO (0.64); Na_2_O (1.32); Cl (0.51); SO_3_ (0.19); Bi_2_O_3_ (8.26); H_2_O+CO_2_ (16.82)

### Cell morphology

Cell morphology was established at 24 h. Cells were fixed for 10 min at room temperature (RT) in 4% paraformaldehyde and 0.1 M phosphate buffer (PB), pH 7.2. After washing the cultures in PBS, they were processed for the direct fluorescence protocol ^14^ using Alexa Fluor 488 phalloidin, a green fluorescent dye conjugated to phalloidin (Molecular Probes, Eugene, USA), and DAPI (Molecular Probes) for labeling the actin cytoskeleton and cell nuclei, respectively. Thermanox^®^ coverslips (Nunc) were affixed to glass slides, after which glass coverslips (12 mm; Thermo Fisher Scientific) were mounted with an antifade kit (Prolong, Molecular Probes) on the Thermanox (Nunc) surface containing the cells. The cells were then examined under a Leica fluorescence microscope (model DMLB, Leica, Wetzlar, Germany) coupled to a Leica DC 300F digital camera.

### Genotoxicity

Genotoxicity was evaluated by the comet assay ^15^ after exposure of the cultures to the different cements. A total of 100 fibroblasts were randomly selected for each experimental group, and their images were analyzed using the Comet Score software program version 2.0.0.38 (TriTek, Sumerduck, USA), based on the migration of DNA fragments. A damage index (DI) was assigned to each comet assay, according to the following formula: DI = (0x n0) + (1x n1) + (2x n2) + (3x n3) + (4x n4), where n = number of cells in each class analyzed (1 to 4). The DI ranged from 0 (no damage, 100 cells x 0) to 400 (maximum damage, 100 cells x 4) ^16^.

### Cell viability

Cell viability analysis was performed at 24 and 72 h by the MTT colorimetric assay [3-(4,5-dimethylthiazol-2-yl)-2,5-diphenyltetrazolium bromide, (Sigma-Aldrich, St. Louis, USA) as described elsewhere ^5^. Briefly, MTT solution (5 mg/mL) was added to the culture medium at 10% of the total volume (final concentration of 0.5 mg/mL), and the cells were maintained in a humidified environment at 37°C with 5% CO_2_ and 95% atmospheric air for four h. The solution was removed, and acidic isopropyl alcohol (0.04 M HCl in 2-propanol; Sigma-Aldrich) was added (1 mL/well). The plates were shaken for 5 min, and the solution absorbance was detected at 570 nm using an Epoch 2 microplate spectrophotometer (BioTek Instruments, Winooski, USA).

### Gene expression

After 72 hours of exposure to different cements, the culture medium was removed from the wells, and Trizol LS reagent (Invitrogen) was added at room temperature (approximately 25°C) for 5 minutes with pipette agitation. Total RNA extraction was performed using the SV Total RNA Isolation System kit (Promega) according to the manufacturer's specifications. Next, total RNA was quantified and converted into cDNA using the High-Capacity cDNA Reverse Transcription Kit (Applied Biosystems, Foster City, CA, USA), following the manufacturer’s instructions. For the real-time PCR reaction, the PowerUp SYBR Green Master Mix for qPCR (Applied Biosystems) and the StepOne System (Applied Biosystems) were used. The reactions were performed in triplicate, with a final volume of 10 μL containing 1 g of cDNA according to the manufacturer’s specifications. Gene expression of type I collagen (COL-1), tumor necrosis factor alpha (TNF-α), interleukin 1 (IL-1), interleukin 6 (IL-6), and metalloproteinase 9 (MMP-9) was evaluated. The results were normalized to the internal control of β-actin (ACTB) and calibrated against the control group. The data are expressed as relative expression levels, calculated using the comparative 2^-ΔΔCt^ method ^17^. The primer sequences used were: COL-1: forward 5’-GCTCCTCTTAGGGGCCACT-3’, reverse 5’-CCACGTCTCACCATTGGGG-3’; TNF-α: forward 5’-AGGCCTTGTGTTGTGTTTCCA-3’, reverse 5’ATGGGGGACAGCTTCCTTCTT3’; IL-1β: forward 5’-GCTACCTGTGTCTTTCCCGT-3’, reverse 5’-CATCTCGGAGCCTGTAGTGC-3’; IL-6:forward 5’- CAATGGCAATTCTGATTGTAT G-3’, reverse 5’-AGGACTCTGGCTTTGTCTTTC-3’; MMP-9: forward 5’-TCCTACTCTGCCTGCACCACTAAAG-3´, reverse 5’-CTGTACCCTTGGTCTGGACAGAAAC-3´; and ACTB: forward 5’-CTCTGGCTCCTAGCACCATGAAGA-3’; reverse 5’- GTAAAACGCAGCTCAGTAACAGTCCG-3’.

### Statistical analysis

The assays were performed with n = 4 technical replicates per group for morphology, genotoxicity, and viability. RT-qPCR was conducted in technical triplicate using cDNAs from 2 independent experiments. Quantitative data were analyzed using the non-parametric Kruskal-Wallis test, followed by the Student-Newman-Keuls post-hoc test when appropriate (α = 0.05), using SigmaPlot (Systat Software, San Jose, USA).

## Results

### Effect of calcium aluminate cement on cell morphology

**Figure 2:**
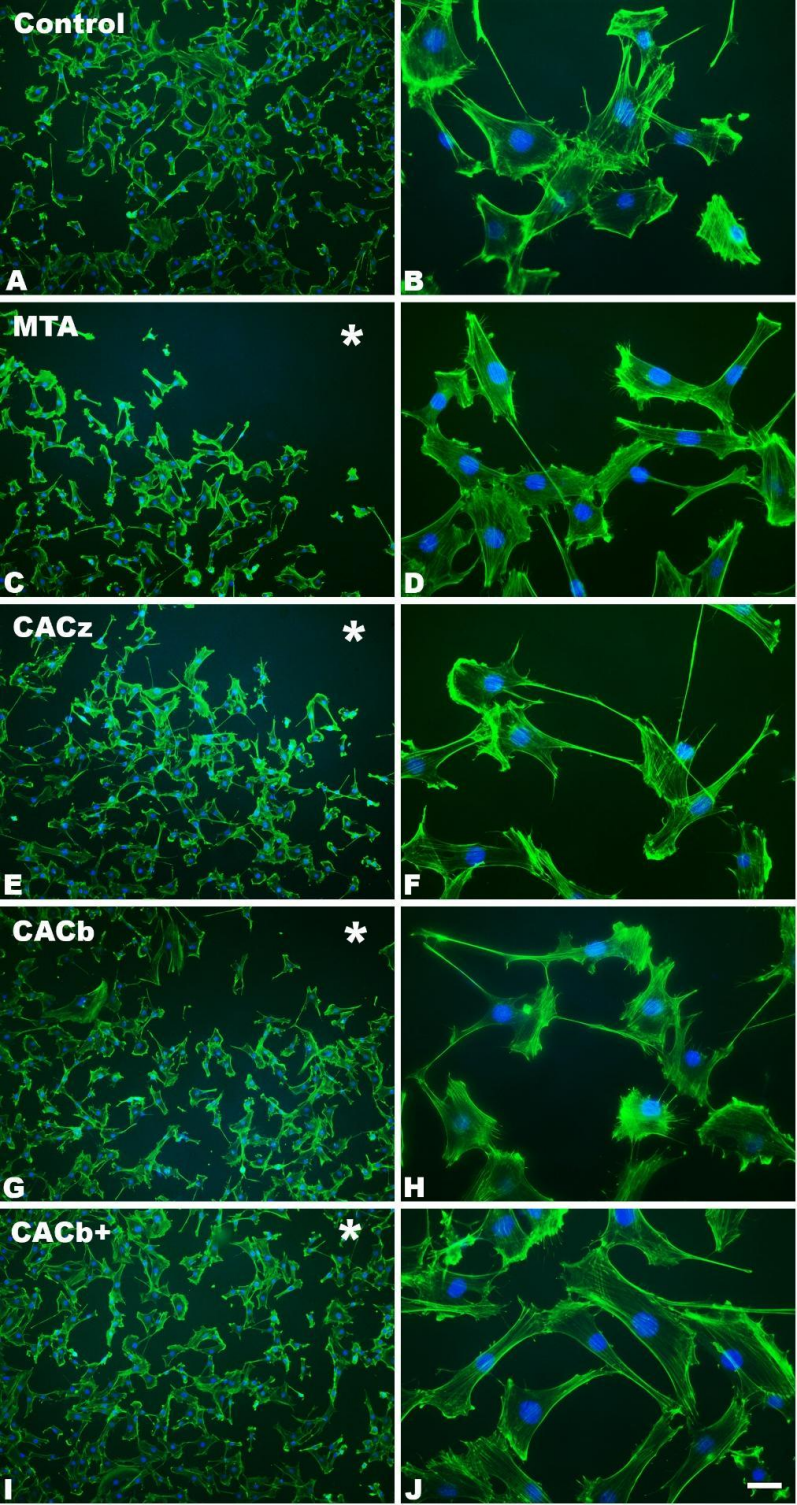
Direct fluorescence of fibroblastic lineage 3T3-L1 cultured after exposure to MTA (Figure 2C-D), CACz (Figure 2E-F), CACb (Figure 2G-H), and CACb+ (Figure 2I-J), at 24 hours. The control group is represented as A-B. Green fluorescence reveals the actin cytoskeleton, and blue fluorescence indicates cell nuclei. Scale bar = 100 µm

At 24 h, the cells were adhered and widely spread in all the experimental groups (Figure 2). Although all groups showed high cell density (Figure 2A-J), a central area devoid of cells was observed in cultures exposed to MTA (Figure 2C-D), CACz (Figure 2E-F), and CACb (Figure 2G-H), in a region that coincided with a greater proximity to the samples (note the asterisks in Figures 2C, E, and G). This area was not evident in the cultures grown in the presence of CACb+ (Figure 2I-J).

### Evaluation of the genotoxic potential of calcium aluminate cement

At 24 h, the genotoxicity assay revealed statically similar DI values in fibroblasts exposed to the control [136 (134-144)], MTA [99 (90-100)], CACz [96 (78-111)], CACb [114 (113-124)], and CACb+ [111 (106-126)] (median, 25% percentile and 75% percentile, respectively; p>0.05), thus indicating low genotoxic potential for all the cements evaluated.

### Effect of calcium aluminate cement on cell viability

After 24 hours, no significant changes in cell viability were observed among the groups, regardless of whether they were exposed to the cements (p = 0.054; Table 2). At 72 h, a significant increase in cell viability was observed for fibroblasts exposed to CACb+, compared with the other groups (Kruskal-Wallis, p=0.012; multiple comparisons p<0.05, Table 2). The other comparisons showed no statistically significant differences (p>0.05; Table 2), including MTA vs CACz (p=0.0566), MTA vs CACb (p=0.9247), and CACb vs CACz (p=0.2267).

**Table 2:** Median values (25% percentile - 75% percentile) of cell viability in fibroblast cultures exposed to MTA and different CAC cement preparations for 24 and 72 hours. Values are expressed as the percentage of absorbance relative to the mean of the control.

		Control	MTA	CACz	CACb	CACb+
Cell viability	24 h	99.1 (94.4-106.6)	106.2 (94.4-107.1)	92.5 (89.0-96.7)	103.8 (93.6-117.9)	103.2 (97.37-107.7)
	72 h	99.9 (97.6-102.5)	108.4 (104.0-109.6)	81.30 (74.5-97.3)	99.2 (98.6-103.8)	148.32 (142.1-158.3)*

Asterisk indicates statistical difference among groups in the same line (n = 4 from technical replicates; Kruskal-Wallis/SNK, p <0.05).

### Effect of different calcium aluminate cement preparations on gene expression of COL-1, IL-1β, IL-6, TNF-α, and MMP-9

After 72 hours, similar gene expression levels of COL-1, IL-1β, and TNF-α were observed in all experimental groups (p > 0.05; Table 3). In addition, all cements upregulated IL-6 gene expression compared with the control (Kruskal-Wallis, p = 0.0007; multiple comparisons, p < 0.05; Table 3). Similar IL-6 gene expression levels were observed in MTA and CACz (p = 0.8188), which were significantly higher than those observed in CACb (p = 0.039) and CACb+ (p = 0.0066; Table 3). MMP-9 gene expression was the highest for CACb+ among all the groups, whereas CACz showed the lowest levels of MMP-9 gene expression (Kruskal-Wallis, p = 0.00007; multiple comparisons p < 0.05; Table 3).

**Table 3:** Median values (25% percentile - 75% percentile) of the relative gene expression values of type I collagen (COL-1), interleukin 1 beta (IL-1β), interleukin 6 (IL-6), tumor necrosis factor alpha (TNF-α), and matrix metalloproteinase 9 (MMP-9) at 72 hours in fibroblast cells, whether exposed or not exposed (control) to different endodontic cements. Levels of expression of the target gene were normalized by the endogenous β-actin control and calibrated by the values obtained in the control group (adopted as 1).

	Control	MTA	CACz	CACb	CACb+
COL-1	1.02(0.97-1.06)	0.85(0.72-0.98)	0.86(0.84-0.91)	0.94(0.87-1.00)	0.91(0.87-0.94)
IL-1β	1.05(0.92-1.10)	1.85(0.70-3.03)	0.92(0.82-1.88)	0.81(0.74-1.14)	1.07(0.73-2.25)
IL-6	1.01(0.95-1.09)	4.75(4.52-4.96)**	4.13(3.32-4.91)**	2.92(1.90-3.90)*	2.69(1.78-3.43)*
TNF-α	1.04(0.93-1.20)	0.92(0.67-1.18)	1.45(1.06-2.19)	0.82(0.63-0.86)	1.07(0.95-1.11)
MMP-9	0.98(0.92-1.05)***	0.74(0.64-0.86)*	0.59(0.51-0.66)	0.87(0.81-0.92)**	1.37(1.17-1.39)****

Asterisks indicate statistical difference among groups in the same line (n = 6 from 2 independent experiments; Kruskal-Wallis/SNK, p <0.05).

## Discussion

The null hypothesis of this study, that different calcium aluminate cement (CAC) formulations would not affect fibroblast viability, genotoxicity, or the expression of proinflammatory markers, was rejected. The results demonstrated that variations in the cement composition, particularly the proportion of calcium chloride and the use of bismuth oxide, significantly influenced cellular responses, including cell viability and the modulation of inflammatory gene expression.

This study was conducted based on the premise that, although CAC has shown promising physicochemical and biological properties, there are critical gaps in the literature regarding its genotoxicity and its capacity to modulate inflammatory processes, especially when modified with commonly used additives such as bismuth oxide and calcium chloride. These aspects are highly relevant since endodontic cements remain in close contact with periapical tissues and must not induce genotoxic effects, exacerbate inflammatory responses, or impair tissue healing. In fact, bismuth-containing formulations have been reported to display variable cytocompatibility, with some studies showing the absence of genotoxicity and cytotoxicity in fibroblasts ^9^. In contrast, others have demonstrated reduced cell viability, alterations in gene expression, and oxidative stress-mediated toxicity (10-12). Additionally, considering the limitations associated with calcium silicate-based cements, including long setting time, handling difficulties, and potential for discoloration, the development of CAC-based alternatives with enhanced biological performance and favorable handling properties represents an innovative and clinically relevant approach. The present findings contribute novel insights into how specific modifications in CAC formulations influence fibroblast behavior, offering critical information for guiding the development of safer and more effective endodontic materials.

Research into the biocompatibility of endodontic materials has received significant attention in dentistry because endodontic compounds can damage surrounding tissues, ultimately affecting the tissue renewal process and leading to the development and/or maintenance of exacerbated inflammatory reactions ^18^. Thus, considering that most of these materials come in contact with living oral tissues, *in vitro* studies evaluating biocompatibility are fundamental. Given that cytotoxicity in cultured cells often correlates with adverse biological responses *in vivo*, *in vitro* studies remain a fundamental step in evaluating the safety of these materials.

Previous studies have shown that CAC exhibits the same satisfactory physicochemical properties as those that allow MTA to be applied in different endodontic therapy procedures ^2^ ^,^ ^4^. To optimize the CAC formulation, an alternative composition was developed with enhanced Ca²⁺ release and suitable radiopacity, utilizing 10% CaCl₂ and 25% Bi₂O₃. The experimental cement showed promising results in both dental and periapical tissue cell lines ^5^ ^,^ ^6^, particularly because it proved less cytotoxic than MTA and favored the deposition of mineralized tissue. Nevertheless, an endodontic cement should also help resolve inflammatory processes. Thus, the present study aimed to determine the genotoxic potential of CAC formulations and their effects on the expression of inflammatory markers in fibroblast cell cultures.

Genotoxicity assays are crucial in dental materials research because they are considered reliable indicators of carcinogenicity ^19^. Ribeiro et al. ^20^ validated the comet assay, also known as single-cell gel electrophoresis, and considered it an adequate method to investigate genotoxicity; it has since become one of the most widely used methods in dental material research. The comet assay was used in the present study to evaluate the genotoxic potential of CAC in comparison with that of MTA. The results showed that fibroblasts exposed to both CAC preparations and MTA for 24 h exhibited a DNA DI similar to or lower than that of the control group, thus indicating a low risk of damage to DNA integrity. Additionally, MTA is non-cytotoxic ^18^ and non-genotoxic in previous studies ^19^ ^,^ ^21^.

*In vitro*, CAC with zinc oxide was not cytotoxic in fibroblast cultures ^22^. In light of these scientific findings, this study assessed the impact of CAC preparations containing bismuth oxide and calcium chloride on the morphology and cellular viability of fibroblasts exposed to various cements. After 24 hours of exposure, the evaluated groups showed no significant changes in cell viability. However, cell morphology analysis revealed lower cell density in areas close to the cement for the MTA, CACz, and CACb groups. However, fibroblasts exposed to CACb+ showed significantly higher cell viability at 72 h compared to the other groups. Cervino et al. ^23^ suggested that MTA is not an inert material; instead, it can release calcium ions when in contact with human tissues, thus favoring cell migration and proliferation, and promoting an antibacterial environment. A study evaluating human fibroblast morphology in contact with this cement revealed that a small number of viable cells remained adherent to the material, and that some of these cells exhibited morphological alterations ^24^. Conversely, the increase in cell viability after exposure to the CACb+ group is consistent with the results observed in osteoblast cell cultures ^5^ and probably relates to the higher levels of calcium released by this cement at 72 h ^25^, levels that could contribute to stimulating cell proliferation.

In the present study, cell morphology images revealed a reduction in cell density in cultures exposed to MTA; however, this did not translate into a significant reduction in cell viability compared with the control group at any of the evaluated time points. We acknowledge that the direct contact of the cement surface with the cultured cells may have influenced local effects, including cell morphology in areas adjacent to the material; however, this methodology was intentionally chosen to simulate a worst-case scenario of direct exposure, which is relevant for endodontic cements placed in proximity to periapical tissues. In addition, the use of eluates or cement extracts was considered but excluded from this stage of the study, as the primary objective was to assess the direct effects of the tested materials on an attached fibroblast monolayer.

In *in vivo* studies, CAC with zinc oxide produced a smaller inflammatory reaction than MTA and was biocompatible when applied to rat subcutaneous tissue ^26^. Cytokines such as IL-1, IL-6, and TNF-α send modulatory stimuli to different cells of the immune system, exhibiting biological activities that include the stimulation of lymphocyte, neutrophil, and macrophage proliferation, as well as increased chemotactic and phagocytic activities. Additionally, they favor hematopoiesis and influence specific immune responses ^27^. Bearing this in mind, the present study investigated the effects of various CAC formulations and MTA on the gene expression of both type 1 collagen and inflammation markers (TNF-α, IL-1β, IL-6, and MMP-9) in fibroblast cell cultures.

The expression levels of COL-1 and IL-1β in cell cultures exposed to MTA, CACz, CACb, and CACb+ were similar to those observed in the control cultures. IL-6 gene expression, however, was upregulated in cell cultures grown in the presence of all cements compared to that detected in the control cultures. IL-6 plays a key role in acute inflammation and is the primary inducer of C-reactive protein, fibrinogen, and serum amyloid A protein, as well as other factors ^28^.On the other hand, it is also involved in mesenchymal vascular cell recruitment and neo-angiogenesis *in vivo* ^29^ and in inducing fibroblast proliferation ^30^.

In this study, the variation in the calcium chloride proportion of CAC influenced not only cell viability but also the expression of inflammatory markers in fibroblasts. Although not statistically significant, a trend toward TNF-α downregulation was observed in the CACb group, which contained bismuth oxide and a lower calcium chloride concentration. In contrast, the CACb+ group, containing bismuth oxide and a higher proportion of calcium chloride, showed the highest MMP-9 expression levels, in addition to increased cell viability. A previous study conducted by our research group demonstrated that CACb+ promotes a greater release of calcium ^5^, an ion that modulates MMP expression and activation, including MMP-9 ^31^. MMPs are a group of calcium- and zinc-dependent endopeptidases responsible for the degradation of collagen and other extracellular matrix (ECM) components by selective specificity. MMP-9 expression is greater in both inflammatory pathologies and the tissue healing process, and plays a crucial role in modulating immune cell function ^32^. MMP-9 is secreted by a large number of cell types (including neutrophils, macrophages, and fibroblasts), and degrades ECM in pathological and physiological conditions, with subsequent activation of major proangiogenic and growth factors, such as vascular endothelial growth factor and fibroblast growth factor-2 ^33^. Considering this, the increased fibroblast proliferation observed in the CACb+ group may be attributed to calcium-mediated upregulation of MMP-9 expression, potentially enhancing the tissue repair process *in vivo*.

Taken together, the findings from the cytotoxicity, genotoxicity, and gene expression analyses demonstrate that the biological behavior of CAC formulations is directly influenced by their chemical composition, particularly the proportion of calcium chloride and the presence of bismuth oxide. The absence of genotoxic effects across all groups indicates that these materials do not compromise DNA integrity, supporting their biological safety ^9^. However, the cytotoxicity and gene expression results reveal distinct patterns of cellular response depending on the formulation. While CACb+ was associated with enhanced cell viability and increased MMP‑9 expression-suggesting a potential role in stimulating extracellular matrix remodeling and tissue repair-formulations with lower calcium chloride (CACb and CACz) exhibited reduced cell density and did not promote MMP‑9 upregulation. Additionally, the consistent upregulation of IL‑6 across all cement groups highlights a standard early inflammatory stimulus, which may play a dual role in both initiating immune responses and promoting angiogenesis and fibroblast proliferation ^34^. These complementary results suggest that while all tested cements are biocompatible at the level of DNA integrity, their capacity to modulate cell proliferation and inflammation is composition-dependent, with CACb+ demonstrating the most favorable profile in terms of supporting cell viability and potentially enhancing tissue repair mechanisms.

Despite these relevant contributions, this study has some limitations. The *in vitro* approach, while essential for preliminary biological screening, does not fully replicate the complexity of the *in vivo* environment, where interactions with immune cells, blood flow, and extracellular matrix components can significantly influence material behavior ^35^ ^,^ ^36^. Furthermore, the use of a single fibroblast cell line limits the extrapolation of these findings to other relevant cell types involved in tissue repair, such as macrophages, stem cells, and endothelial cells ^37^. The focus on a selected panel of proinflammatory genes, although informative, does not encompass the broader spectrum of molecular pathways involved in inflammation resolution and tissue regeneration.

Future studies should include *in vivo* evaluations to confirm the biocompatibility and bioactivity of these formulations under physiological conditions, as well as explore additional biomarkers related to oxidative stress, angiogenesis, and chronic inflammation. Investigating the interaction of these materials with immune cell populations, particularly macrophages, could provide further insights into their role in modulating the inflammatory response and supporting tissue repair. Moreover, optimizing the balance between radiopacifier content and calcium release remains a relevant goal for enhancing the biological and mechanical performance of CAC-based materials in clinical applications.

## Conclusion

The results of this study indicated that the different CAC formulations containing calcium chloride and radiopacifiers were neither genotoxic nor cytotoxic in fibroblast cultures, thereby preserving cell viability and supporting the expression of fibroblast phenotypic markers. In addition, all cements modulated the inflammatory process by upregulating IL-6 gene expression. However, only CACb+ promoted fibroblast culture proliferation, associated with the upregulation of MMP-9 expression. The expression levels of COL-1 and IL-1β remained unchanged across all groups, and CACb downregulated TNF-α expression. These findings indicate that the biological response of fibroblasts is composition-dependent, particularly influenced by the concentration of calcium chloride.
